# Lactate infusion increases circulating pro-brain-derived neurotrophic factor levels in humans

**DOI:** 10.3389/fncel.2025.1644843

**Published:** 2025-09-23

**Authors:** Julia Röja, Nicolas Fiori Ameller, Jonathan Grip, William Apró, Marcus Moberg

**Affiliations:** ^1^Department of Physiology, Nutrition and Biomechanics, The Swedish School of Sport and Health Sciences, Stockholm, Sweden; ^2^Department of Clinical Science, Intervention and Technology, Karolinska Institutet, Stockholm, Sweden; ^3^Department of Physiology and Pharmacology, Karolinska Institutet, Stockholm, Sweden

**Keywords:** BDNF, pro-BDNF, lactate infusion, human, skeletal muscle, cortisol, BDNF polymorphism, fiber type

## Abstract

Brain-derived neurotrophic factor (BDNF) is a key mediator of neuroplasticity and responsive to acute physical exercise, providing a link between exercise and brain health. Lactate, a metabolite related to exercise, has been proposed as a potential mediator of the BDNF exercise response; however, lactate’s role in isolation has not yet been determined. To investigate this, 18 young, healthy volunteers (50% female) were recruited to donate blood and muscle before, during, and after a 1-h venous infusion of sodium lactate (125 μmol × kg FFM^–1^ × min^–1^) or isotonic saline. Muscle and blood samples were collected during 120 min of recovery from the infusion. Samples were analyzed for pro-BDNF and mBDNF using enzyme-linked immunosorbent assay and immunoblotting. The participants reached a peak plasma lactate level of 5.9 ± 0.37 mmol × L^–1^ in the lactate trial (*p* = 0.0002 vs. Pre). Plasma pro-BDNF levels increased 15 min post lactate infusion and stayed elevated throughout the recovery (55%–68%, *p* < 0.0286 vs. Saline) while plasma and serum levels of mBDNF showed no significant change (*p* > 0.05 vs. Saline). Muscle pro-BDNF levels were also unaltered by the lactate infusion (*p* > 0.05 vs. Saline); however, the expression of pro-BDNF correlated with the proportion of type I muscle fiber area (fCSA%) of the participants (*n* = 18, *r* = 0.6746, *p* = 0.0021). Muscle levels of the mBDNF isoform were non-detectable. In conclusion, these results suggest that lactate in isolation affects circulatory pro-BDNF, but not mBDNF levels. This implies that lactate may partly mediate the exercise response of pro-BDNF in humans.

## Introduction

Mature brain-derived neurotrophic factor (mBDNF) and its uncleaved precursor protein pro-BDNF are broadly acknowledged as central factors for control of neuroplasticity, and thus brain health (Park and Poo, 2013; Brigadski and Lessmann, 2020). A key mechanism for mBDNF-mediated brain plasticity involves binding and stimulation of the tropomyosin receptor kinase B (TrkB) leading to enhanced neuronal survival, synaptogenesis and long-term potentiation (Hampstead, 2015; Lin et al., 2018). In a yin-yang-like fashion, pro-BDNF binding to its preferred receptor is suggested to primarily initiate pro-apoptotic signaling pathways, e.g., inducing long-term depression (Teng et al., 2005; Foltran and Diaz, 2016). However, both growth-promoting and pruning signals are important for proper brain development and neuroprotection (Eggert et al., 2021). Altered BDNF levels in the central nervous system (CNS) and circulation are implicated in various neurodegenerative diseases (Ng et al., 2019; Tapia-Arancibia et al., 2008) and a shift in the ratio between the isoforms toward more pro-BDNF signaling and/or reduced mBDNF-signaling has been proposed to be indicative of different neurological conditions, including Alzheimer’s disease (Fleitas et al., 2018; Li et al., 2023). For instance, chronic mBDNF injections have been demonstrated to positively affect amyloid precursor protein (APP) processing in rodents, reducing the accumulation of amyloid beta (Aβ) peptides (Baranowski et al., 2023), a key aspect in the progression of Alzheimer’s disease (Eggert et al., 2021).

Pro-BDNF can be cleaved, both intra- and extracellularly to the mature form and both isoforms are highly present in the CNS (Binder and Scharfman, 2004; Foltran and Diaz, 2016) but also appear in significant levels in the circulation (Radka et al., 1996; Klein et al., 2011), where especially mBDNF is predominantly bound to platelets (Fujimura et al., 2002). Both BDNF proteins also circulate freely, and this unbound pool is proposed to represent the bioavailable form that is free to bind to its receptors (Fujimura et al., 2002). Although termed brain-derived, BDNF is expressed in various tissues in the periphery, including the metabolically active skeletal muscle. Both BDNF mRNA and BDNF protein have been identified in murine (Koliatsos et al., 1993; Liem et al., 2001; Mousavi et al., 2004) and human skeletal muscle tissue (Matthews et al., 2009; McKay et al., 2020; Máderová et al., 2019), and the levels in rats tend to increase with physical exercise (Matthews et al., 2009; McKay et al., 2020). In humans, quantifiable data and data that distinguish between the mature- and pro-form are limited; however, we recently found that pro-BDNF, and not mBDNF, was highly expressed almost exclusively in the slow oxidative type I fibers of skeletal muscle tissue from healthy adults, and that the muscle levels of pro-BDNF were augmented by exercise (Edman et al., 2024). These data collectively highlight that muscle fiber type composition is an aspect to consider when studying BDNF-metabolism in humans, particularly in relation to exercise.

Circulating mBDNF levels have been demonstrated to increase with both acute resistance- and aerobic-type exercise (Dinoff et al., 2017; Marston et al., 2017) and following aerobic exercise training (Dinoff et al., 2016). Furthermore, we recently showed that both resistance and cycling exercise can induce a significant increase in pro-BDNF levels in young, healthy individuals, (Edman et al., 2024; Tarassova et al., 2025) and that pro-BDNF is released from the exercised muscle (Tarassova et al., 2025). This may however be exercise-modality specific, as running exercise, by contrast, did not cause an increase in pro-BDNF (Edman et al., 2025). Regardless, significant inter-individual variability is often seen in exercise-induced circulatory BDNF increase (Edman et al., 2025; Tarassova et al., 2025), which may be partly explained by a single-nucleotide polymorphism on the BDNF gene, resulting in a valine (Val) to methionine (Met) substitution at codon 66 (Val66Met). In an early study with human participants, it was proposed that the possession of the Met allele reduced the activity-dependent secretion of BDNF on the cellular level (Egan et al., 2003). Rodent models using voluntary wheel running further show either a similarly clear effect (Ieraci et al., 2016), or a less evident effect where there may be a sex and allele interaction with exercise (Jaehne et al., 2023).

In addition to changes in circulating mBDNF in response to acute physical exercise, exercise also leads to transient intensity-dependent increases in cortisol levels (Hill et al., 2008). Like BDNF, cortisol has major implications in the regulation of neuroplasticity (Gray et al., 2013) and over the past decades, evidence has gathered for an interaction between BDNF and cortisol at various levels in the CNS (de Assis and Gasanov, 2019; Tsimpolis et al., 2024). Furthermore, it has been suggested that the BDNF response (unspecified isoform) to acute exercise is intensity-dependent, potentially mediated by blood levels of lactate (Ferris et al., 2007; Marston et al., 2017). Several reports suggest a positive correlation between both serum- (Ferris et al., 2007; Marston et al., 2017) and plasma (Gibbons et al., 2023) levels of BDNF (unspecified isoform) and blood levels of lactate following physical exercise in humans. Adding to this, we recently showed that acute resistance exercise with a simultaneous lactate infusion induced a greater increase in plasma levels of mBDNF compared to resistance training alone (Edman et al., 2024). Moreover, lactate’s role as a regulator of BDNF metabolism during exercise has gained mechanistic support in a mouse model, where inhibition of lactate transporters (monocarboxylate transporters; MCT) 1/2 eliminated the exercise-mediated increase in hippocampal BDNF expression (El Hayek et al., 2019). Lactate levels were further modulated at rest via repeated lactate injections, which increased lactate levels in the mice’s hippocampus and enhanced long-term BDNF mRNA expression and protein levels (El Hayek et al., 2019). In a recent study, acute lactate injections did not alter BDNF gene expression or protein content in the mice’s hippocampus or prefrontal cortex (Moberg et al., 2024), highlighting a potential distinction between the effects of chronic and acute lactate elevations on BDNF expression in the CNS. In addition to the BDNF-stimulating potential of lactate, it is also worth noticing that lactate is suggested to improve brain plasticity via other mechanisms, potentially via activation of the lactate sensitive G-protein coupled receptor 81, also known as HCAR1 (Yang et al., 2014; Herrera-López and Galván, 2018; Herrera-López et al., 2020). Of further relevance for brain health, lactate administration has been demonstrated to alter the activity of amyloid precursor protein processing enzymes ADAM10 and BACE1, which are under BDNF control (Moberg et al., 2024).

In human exercise studies, acute changes in circulating BDNF levels have been frequently used as a proxy marker for acute CNS synthesis and release (Walsh and Tschakovsky, 2018); however, recent findings provide indications that the acute exercise-induced increase in serum mBDNF might solely be a result of activity-dependent splenic platelet release (Tarassova et al., 2025). Here, we removed the exercise stimulus to isolate the potential effects of lactate on BDNF levels. At this point, it is uncertain whether lactate can affect plasma and serum mBDNF levels with no simultaneous exercise stimulus and whether lactate influences acute circulatory and/or muscle pro-BDNF levels in humans. Hence, the present study aimed to increase systemic lactate levels in humans at rest and examine how this physiological alteration affected circulatory plasma and serum, as well as muscle levels of the pro- and mBDNF isoforms. We further measured circulating cortisol and considered the potential moderating effects of muscle fiber type and genotype on BDNF levels in the circulation and skeletal muscle in response to lactate infusion.

## Materials and methods

### Ethical approval

The study protocols were approved by the Swedish Ethical Review Authority (diary numbers 2021-02993 and 2022-01369-02), and except for being registered in a database, the study was executed in agreement with the ethical standards defined in the Declaration of Helsinki.

### Participants

Eighteen healthy human volunteers (self-reported biological sex; female/male, 50% females) were recruited for this study, twelve participants to an experimental group (50% females) and six to a control group. To be considered for enrollment, volunteers were required to be 20–40 y/o, non-smokers and free from medical conditions and medications that could potentially influence the study outcomes (e.g., antidepressants) as well as not performing more than low levels of physical exercise. All participants were regarded as untrained to recreationally trained, performing zero to two recreational training sessions per week, involving resistance- and aerobic-type exercises, yoga, and various sports activities, e.g., badminton and football. Participant characteristics for the experimental and the control group are presented in Table 1. All participants provided oral and written informed consent prior to enrollment.

**TABLE 1:** Subject characteristics in the experimental and control group.

Characteristic	Experimental group (*n* = 12)	Control group (*n* = 6)
Female/male)^a^	6/6	3/3
Age (years)	28.7 ± 5.0	29.2 ± 2.9^b^
Height (cm)	174 ± 13	170 ± 12^b^
Weight (kg)	67.4 ± 13.7	67.6 ± 12.8^b^
BMI (kg/m^2^)	22.0 ± 1.6	23.3 ± 2.3^b^
FFM (kg)	49.1 ± 11.6	52.3 ± 10.4^b^
Body fat (%)	26.6 ± 4.0	22.3 ± 7.0^b^
VO_2_max (L min^–1^)	2.75 ± 0.65	3.05 ± 0.52^b^
VO_2_max (mL kg^–1^ min^–1^)	40.6 ± 4.6	44.8 ± 3.9^b^
Type I fiber%	41.1 ± 13.2	49.0 ± 13.8^b^
Type I fCSA (μm^2^)	4139 ± 1192	4593 ± 1299^b^
Type I fCSA%	41.0 ± 13.2	50.6 ± 17.0^b^
Val/Met (*n*)	5	2
Val/Val (*n*)	7	4

a Self-reported biological sex (female/male).

b Indicates no significant difference between groups (*p* > 0.05, Welch’s *t*-test; control group *n* = 6 vs. experimental group *n* = 12). BMI, body mass index; FFM, fat-free mass; VO_2_max, maximal oxygen uptake; fCSA, fiber cross-sectional area; fCSA%, percentage fiber type cross-sectional area; Val, valine; Met, methionine; Val/Met, participants expressing the BDNF Val66Met polymorphism. Values are mean ± standard deviation (SD).

### General study design

**FIGURE 1:**
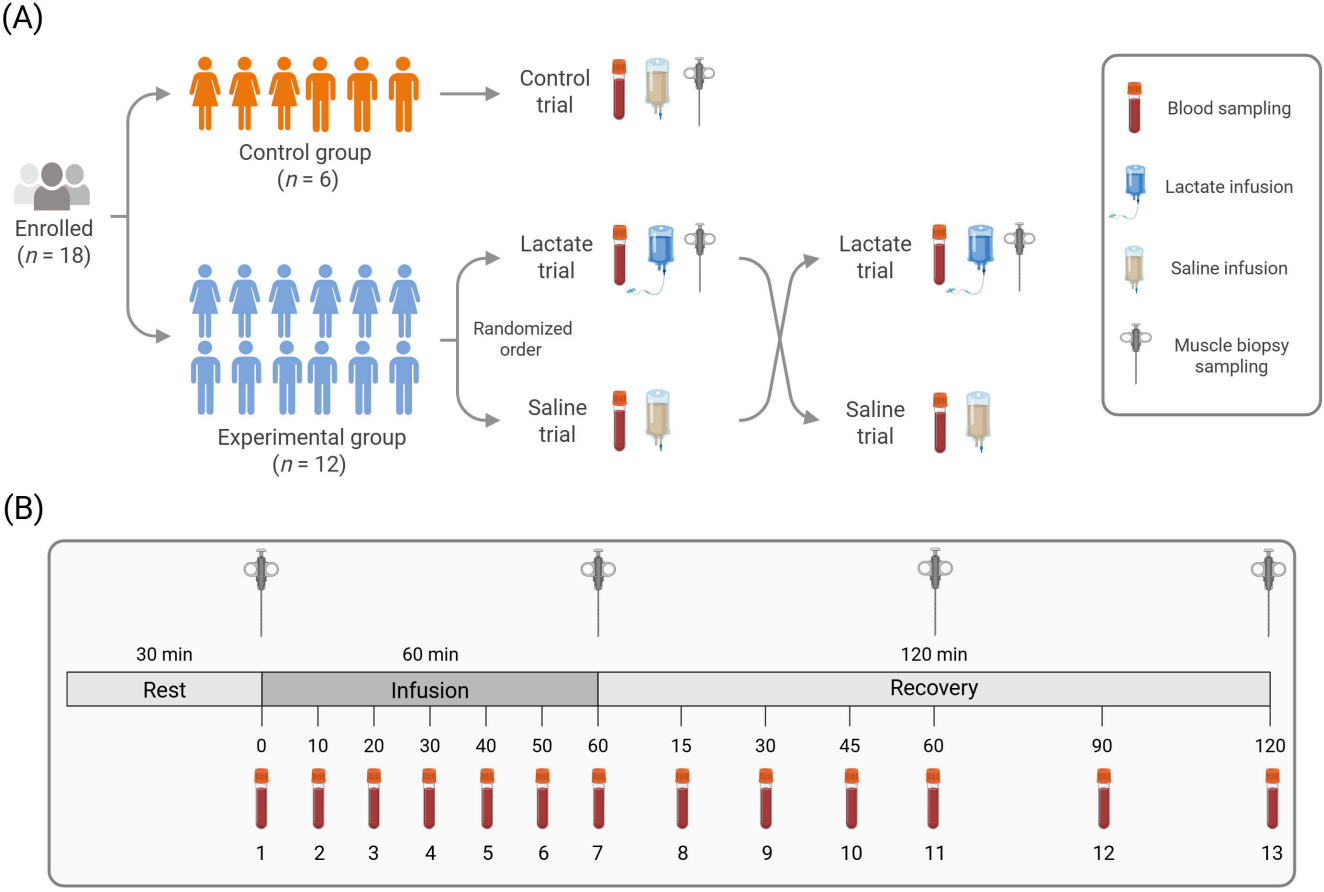
Illustrative overview of the general study design **(A)** and experimental trial protocol **(B)**. **(A)** Of the participants enrolled (50% female), six were assigned to the control group and twelve to the experimental group. The control group participated in one trial and the experimental group in two, in randomized order. **(B)** Biopsy needles indicate muscle biopsy time points. Blood vials indicate blood sampling time points. Blood samples were collected at thirteen time-points in all trials. Muscle biopsies (Lactate and Control trial) were collected at four time points. Illustration created in BioRender.

For all blood parameters, a randomized crossover-controlled design was employed in which each participant in the experimental group rested in a supine position for approximately 4 h on two different occasions while receiving a 60-min venous infusion of either sodium lactate (Lactate trial) or isotonic saline (Saline trial). The two experimental trials were separated by seven to 30 days. Blood was sampled on both occasions. In addition to blood samples, muscle biopsies were taken from participants in the experimental group, but only in the Lactate trial. To reduce the number of muscle biopsies for the participants in the experimental group, a smaller control group was also recruited (*n* = 6). This was warranted out of ethical aspects, especially as there was very little, if any, rationale that a saline infusion and repeated biopsies would induce changes in skeletal muscle relevant to the aims of this study. Each participant in the control group took part in an experimental trial where they donated muscle biopsies while receiving a venous infusion of isotonic saline (Control trial). The muscle data from the Control trial were subsequently used to compare with the muscle-specific response in the Lactate trial of the experimental group. An illustrative overview of the general study design is provided in (Figure 1).

### Enrollment procedure

All volunteers visited the laboratory for an information meeting and health screening to determine eligibility. If criteria were met, volunteers had their maximal oxygen uptake (VO_2_max) determined with a graded exercise test on a treadmill using COSMED Quark CPET breath-by-breath analysis with Omnia software (COSMED The Metabolic Company, Rome, Italy). Volunteers were instructed to refrain from eating within 3 h prior to testing and to arrive well-rested and in good health. The test protocol began with a 5-min warm-up at an individually selected light pace, immediately followed by two 5-min stages at two different moderate intensities (0.5°incline). After a few minutes of active rest, the maximal test began at an individually selected running speed. Treadmill velocity was subsequently increased by 0.5–1.0 km/h every minute until the participant reached their highest sustainable running speed without significant deterioration in running form. Thereafter, the treadmill incline was increased by 1° for 1 min, followed by an additional 0.5° increase for each subsequent minute. The test continued until volitional exhaustion and participants were asked to report their rating of perceived exertion (RPE) using the Borg scale. VO_2_max was accepted as valid if at least two of the following criteria were met: (a) a plateau in VO_2_ despite an increase in workload, (b) RPE ≥ 18, and (c) a respiratory exchange ratio (RER) ≥ 1.1. The highest 45-s average of VO_2_ was considered the participant’s VO_2_max. Enrolled participants came back in a fasted state and had their body composition measured with dual x-ray absorptiometry (DXA) to assess their fat-free mass (FFM). All participants were instructed to avoid increasing their habitual amount of physical exercise throughout the study, and 2 days before each trial participants were told to fully refrain from physical exercise.

### Experimental trials

On the trial days, participants reported to the laboratory at 7:30 AM in an overnight fasted state. After arrival, participants took a supine position and had a 20- or 22-gauge Teflon catheter inserted into the antecubital vein of each forearm, one for infusion and one for repeated blood sampling. Following a 30-min rest, a baseline blood sample was obtained to confirm that fasting glucose, resting lactate, acid-base balance and electrolyte status were within established normal reference values. In the Lactate and Control Trials, a baseline muscle biopsy was then taken from the vastus lateralis muscle. The biopsy procedure was performed as described in Liegnell et al. (2020). In short, samples were taken under local anesthesia (Carbocaine 20 mg, Astra Zeneca AB, Sweden) using a Bergström needle (Stille, Torshälla, Sweden) with applied suction (Evans et al., 1982). After baseline sampling, a venous infusion of sodium lactate (APL, Stockholm, Sweden) or a volume-matched isotonic saline (0.9% sodium chloride) infusion (Fresenius Kabi AB, Uppsala, Sweden) was initiated using an automated infusion pump. The concentration of the sodium lactate solution was 1 M (pH 6.4) and the infusion rate was 125 μmol × kgFFM^–1^ × min^–1^ with a total infusion time of 60 min. Throughout the infusion blood was drawn every 10 min. Immediately following the termination of the infusion, a blood sample was collected, and a second muscle biopsy (Lactate- and Control trial) was taken from the same leg. Thereafter, the participant stayed in a supine position and blood samples were drawn after 15, 30, 45, 60, 90, and 120 min. Blood that was not used for immediate analyses was collected in heparinized- and serum-separating tubes. To prevent between-sample contamination, the catheter used for blood sampling was flushed with a saline solution between each blood sample. In the Lactate- and Control trials, additional biopsies were taken from the same leg at 60- and 120 min post-infusion. An illustrative overview of the experimental trial protocols is presented in Figure 1.

### Whole blood analysis

Whole blood was analyzed for levels of pH, base excess, sodium, potassium and hematocrit (HCT) using the handheld i-STAT1 blood analyzer with EG6 + cassettes (Abbot Laboratories, Chicago, IL, USA). Due to availability reasons whole blood levels of lactate were analyzed with either the Biosen C-line analyzer (EKF-Diagnostics, Cardiff, UK) or the Lactate Scout 4 (EKF-Diagnostics, Leipzig, Germany). Whole blood lactate measurements were primarily used for monitoring infusion progress during the trials. In the Saline trial, whole blood was only analyzed for pH, base excess, sodium, potassium and HCT at baseline, 30 and 60 min after infusion start, and 15-, 30-, 60-, and 120 min after the end of infusion.

### Blood sample preparation

Whole blood sampled in heparinized tubes was stored on ice until centrifuged at 3000 *g* at 4°C for 10 min. The upper- and bottom half of the plasma obtained was then transferred to new separate tubes, the bottom half saved as plasma and the upper half spun again at 3000 *g* at 4°C for 10 min. The top 75% was then collected and stored as platelet-poor plasma (PPP). Serum-separating tubes were kept agitated at room temperature for a minimum of 15 min. After centrifuging at 2000 *g* at 4°C for 10 min serum was transferred to new tubes. All plasma- and serum samples were stored at −80°C until analysis.

### Muscle sample preparation

Immediately after collection, muscle samples were quickly blotted, cleared of blood and snap-frozen in liquid nitrogen before subsequent storage at −80°C. Following lyophilizing, samples were thoroughly dissected free from blood and connective tissue under a light microscope (Carl Zeiss, Germany) and organized into small bundles of fibers. The fiber bundles were then mixed and separated into aliquots. For immunohistochemistry, a piece of the sampled muscle was instantly cleared from blood and connective tissue and placed on a flat surface with the fibers pointing vertically. The sample was then embedded in an O.C.T. medium (Tissue-Tek O.C.T. Compound) and frozen in isopentane pre-chilled in liquid nitrogen. Samples were stored at −80°C until cryosectioning.

### Plasma and muscle lactate analyses

Plasma and muscle lactate levels were analyzed spectrophotometrically (Bergmeyer, 1974) using a microplate reader (Infinate 200 Pro, Tecan, Männedorf, Switzerland). To determine plasma lactate concentrations 10 μl plasma was added to a 48-well microplate. A glycine-hydrazine buffer (pH 8.8) consisting of 0.5 M glycine, 0.4 M hydrazine hydrate, 10 mm ethylenediaminetetraacetic acid (EDTA) and 500 ml distilled water (dH2O) was mixed thoroughly with nicotinamide adenine dinucleotide (NAD) and lactate dehydrogenase (LDH) to form the reaction solution. 770 μl reaction solution was subsequently added to each well, and following a 60 min incubation at room temperature, the absorption of the samples was read at 340 nm, and the concentrations were calculated with the following equation: (Absorbance – Blank) × μl in well (780 μl)/6.22 × μl added sample (10 μl) = mmol/L.

To determine muscle lactate levels, 2 mg of lyophilized muscle was transferred into Eppendorf tubes using a Mettler Toledo XA105 Dual Range Scale (*d* = 0.01 mg/0.1 mg). All samples were homogenized in trichloroacetic acid (TCA) for five min using a BulletBlender™ (Next Advanced, New York, USA) with zirconium oxide beads. Tubes were then centrifuged at 3000 *g* (4°C) for five min after which the solution was transferred to new tubes and mixed with 1 M potassium chloride. Thereafter, 25 μl of the muscle homogenate was pipetted into a 48-well microplate, followed by a 500 μl reaction solution with the same content as described above. Enzymatic reactions were then run for 60 min at room temperature, followed by absorbance readings at 340 nm. Lactate levels were quantified from the absorbance readings using the following equation: (Absorbance – Blank) × μl in well (525 μl)/6.22 × μl added sample (25 μl) × 100/mg muscle × 1.33 = mmol/kg dry muscle. The muscle levels in mmol/kg dry muscle (dry weight; d. w.) were also recalculated to mmol/L intracellular (i.c.) water in agreement with the data from Sjøgaard et al. (1985). For the plasma- and muscle lactate analysis, blanks, controls, and samples were assayed in duplicates, and the average readings were used for calculations.

### Serum cortisol

Serum levels of cortisol were analyzed with the Cortisol ELISA-kit (CO368S) and performed following the directions for analyses provided by the manufacturer (Calbiotech Inc., El Cajon, CA, USA). A Wellwash™ Microplate Washer (Thermo Fisher Scientific, Waltham, MA, USA) was used for all assays and assays were run with standards, controls and samples in duplicates. Each participant’s samples from both trial conditions (Lactate and Saline trial) were analyzed on the same ELISA plate and the intra-assay coefficients of variation (CV%) for all samples were <9% averaging 2.17% ± 1.71% (SD). All ELISA plates, reagents and standards were sourced from a single batch (same lot number).

### Pro and mBDNF analyses with enzyme-linked immunosorbent assay

Plasma levels of pro-BDNF (pg × ml^–1^), PPP-levels of mBDNF (pg × ml^–1^) and serum levels of mBDNF (pg × ml^–1^) were quantified with enzyme-linked immunosorbent assay (ELISA). The Human Pro-BDNF DuoSet ELISA kit (DY3175), in combination with the DuoSet Ancillary Reagent Kit 2 (DY008) from R&D Systems (Minneapolis, MN, USA), was used for pro-BDNF analyses. Assays were performed in accordance with the manufacturer’s instructions apart from a few adjustments. Standards and samples were diluted in sterile PBS, standards with 10% FBS + 0.02% Tween-20 and samples with 1% FBS, and then incubated in the coated plate overnight (16 h) at 4°C. All plasma samples used for pro-BDNF analysis were diluted by 20% in 1% FBS. To quantify mBDNF in PPP- and serum samples the Human Free BDNF Quantikine Immunoassay kit (DBD00) from R&D Systems was used. The assays were run according to the manufacturer’s directions with serum samples diluted 20-fold in the diluent provided with the kit. A Wellwash™ Microplate Washer (Thermo Fisher Scientific, Waltham, MA, USA) was used for all assays and the assays were performed with standards, controls and samples in duplicates. All samples from each individual participant were analyzed on the same ELISA plate for each of the three assays. The mean intra-assay coefficients of variation were 2.1% for pro-BDNF, 2.3% for PPP mBDNF and 2.3% for serum mBDNF. All ELISA components for each of the three assays were from the same production lot.

### Muscle pro and mBDNF analyses with immunoblotting

Immunoblotting was performed in accordance with the procedure described in Liegnell et al. (2020) with antibodies validated by Edman et al. (2024).

### Immunohistochemistry

Cryosectioning and the subsequent immunohistochemical procedures for determination of fiber type composition and fiber cross-sectional area (fCSA) were performed as described previously (Horwath et al., 2020; Horwath et al., 2021a; Horwath et al., 2021b) and the percentage fiber type cross-sectional area (fCSA%) was calculated as explained in Horwath et al. (2021a). The fiber type characteristics for participants in the experimental and the control group are presented in Table 1.

### Genotyping

For genotyping the Val66Met polymorphism in the human BDNF gene, a one-step amplified refractory mutation system (ARMS) polymerase chain reaction (PCR) was performed in agreement with the method outlined in Sheikh et al. (2010) and the PCR amplification run in CFX96 touch real-time PCR detection system (Bio-Rad Laboratories, Sundbyberg, Sweden). Prior to the ARMS-PCR, isolation of total DNA was completed using approximately 3 mg of lyophilized and dissected skeletal muscle tissue. The extraction was performed using the DNeasy Blood and Tissue kit (Cat. No. 69504) from Qiagen (Hilden, Germany) and the spin-column protocol for purification of total DNA from animal tissues. Electrophoresis was run on a 5% TBE polyacrylamide precast gel (Bio-Rad Laboratories, Mississauga, Canada) and then soaked with SYBR*™* Safe DNA gel stain concentrate (Invitrogen, Thermo Fisher Scientific, Carlsbad, CA, USA) diluted in TBE buffer. PCR amplicons were subsequently documented with the ChemiDoc MP Imaging system (Bio-Rad).

### Statistics

The sample size in the experimental group (*n* = 12) was based on power calculations from a pilot experiment in four individuals (data not presented). A minimum sample size of *n* = 8 was estimated based on a statistical power of 0.80 (α = 0.05), using Cohen’s *d* = 0.4, which corresponds to an anticipated mean difference of approximately 50% in mBDNF levels, based on mBDNF data from Edman et al. (2024). To account for potentially smaller effects, four additional participants were enrolled in the experimental group to ensure sufficient power. Data were analyzed using TIBCO Statistical 13 for Windows (TIBCO Software Inc., Palo Alto, CA). The data are presented as mean ± standard deviation (SD). Weighted means were used to replace two missing blood and plasma lactate values before analysis. The normal distribution was assessed with histograms and the Shapiro-Wilk test of normality. Following log transformation of the cortisol data, all variables were considered acceptable for parametric statistics. However, the results present the untransformed means to facilitate the interpretation of the data. A two-way repeated measures analysis of variance (ANOVA; trial × time) was used for the data analysis on circulating levels of plasma- and serum mBDNF, pro-BDNF, and cortisol (2 × 5), plasma lactate (2 × 11), whole blood lactate (2 × 13) as well as sodium, pH, base excess, potassium and HCT (2 × 7). If the ANOVA showed a significant main effect of time or interaction effect, a Tukey’s Honest Significant Difference (HSD) test was performed. Muscle BDNF levels were analyzed using a two-way ANOVA (group × time) with four time points (baseline, post-infusion, and 60- and 120 min post-infusion) to compare the muscle data from the Lactate trial of the experimental group (*n* = 12) with the muscle data from the control groups saline trial (*n* = 6). Correlations between fiber type and muscle pro-BDNF levels as well as between cortisol and plasma mBDNF were calculated with Pearson’s product-moment correlation (r). Welch’s *t*-test was conducted to assess differences in BDNF levels between participants with and without the BDNF Val66Met polymorphism. Additionally, Welch’s *t*-test was used to compare baseline descriptive variables between the experimental and control groups to confirm group homogeneity (Table 1). A significance level of α = 0.05 was employed for all statistical tests.

## Results

### Lactate levels

**FIGURE 2:**
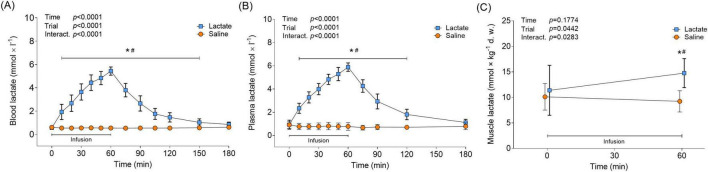
Circulatory and muscle lactate in response to lactate infusion. **(A)** Levels of blood lactate and **(B)** plasma lactate in the Lactate and Saline trial (*n* = 12 vs. 12). **(C)** Levels of muscle lactate (*n* = 12 vs. 6). *p < 0.05 vs. Pre, #*p* < 0.05 vs. Saline. Two-way repeated measures ANOVA, Tukey’s HSD *post hoc*. Error bars show variability as ± 1 standard deviation (SD). Error bars for the lactate trial are shown in bold.

Lactate infusion continuously increased blood and plasma levels of lactate over the 60-min infusion protocol, peaking at 5.4 ± 0.36 and 5.9 ± 0.37 mmol × L^–1^, respectively (Figures 2A, B). Immediately upon infusion cessation in the Lactate trial, blood and plasma lactate decreased from peak levels (*p* = 0.0002; 75 min vs. 60 min) and returned to baseline levels following 120 min of recovery (180 min vs. Pre, *p* > 0.8; Figures 2A, B). Blood and plasma lactate levels remained unaltered in the Saline trial throughout the protocol (*p* > 0.05 vs. Pre; Figures 2A, B). At baseline, average plasma lactate levels were 60% greater than mean whole blood levels; however, the difference was reduced to 10% when peak lactate levels from the infusion were reached. Lactate infusion increased muscle lactate levels by 29%, from 11.4 ± 4.88 to 14.8 ± 2.86 mmol × kg^–1^ d. w. (Figure 2C), or 3.07 to 3.98 mmol/L i.c. water (*p* = 0.0044 vs. Pre). Muscle lactate levels remained unchanged with saline infusion (*p* = 0.5488 vs. Pre; Figure 2C).

### Blood pH, ions, and serum cortisol

**FIGURE 3:**
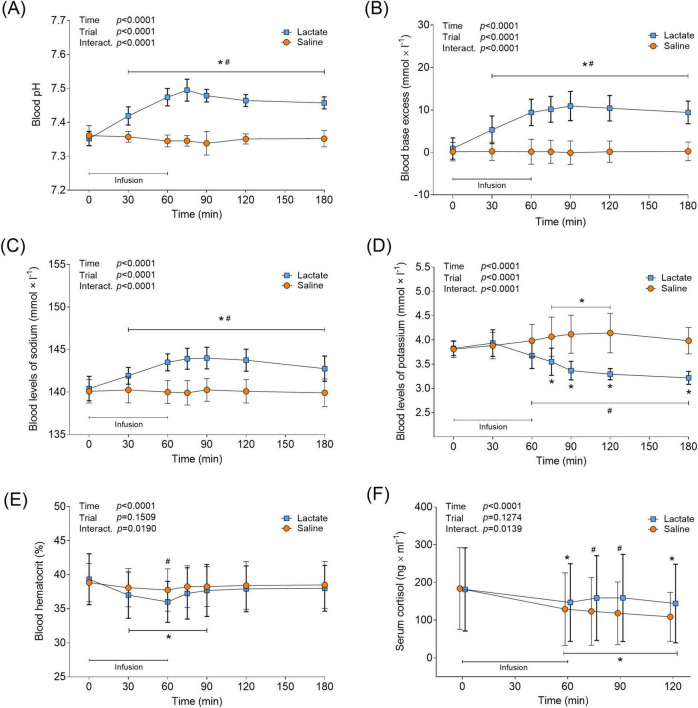
Blood parameters and serum cortisol in response to lactate infusion. **(A)** Blood pH, **(B)** blood base excess (BE), **(C)** blood levels of sodium, **(D)** blood levels of potassium, **(E)** blood hematocrit and **(F)** serum cortisol levels (*n* = 12 vs. 12). *p < 0.05 vs. Pre, #*p* < 0.05 vs Saline. Two-way repeated measures ANOVA, Tukey’s HSD *post hoc*. For clarity, panel **F** illustrates the raw serum cortisol data, although statistical analysis was performed on log transformed values. Error bars show variability as ± 1 standard deviation (SD). Error bars for the lactate trial are shown in bold.

Lactate infusion had an alkalizing effect on blood, increasing pH from baseline levels (7.35 ± 0.02) to 30 min into the infusion, and then remaining elevated throughout the trial (7.42 ± 0.03–7.49 ± 0.03, *p* < 0.0001 vs. Pre; Figure 3A), while saline infusion had no effect on pH (*p* > 0.0547 vs. Pre; Figure 3A). The blood base excess followed the changes in pH for both trials (Figure 3B). Blood levels of sodium increased 30 min into the lactate infusion and remained elevated throughout the trial (141.9 ± 1.0–144.0 ± 1.28 mmol × L^–1^, *p* < 0.0001 vs. Pre, Figure 3C). Saline infusion did not affect blood sodium levels in the Saline trial (*p* > 0.9848 vs. Pre, Figure 3C). 15 min after the end of the lactate infusion (min 75) and throughout the recovery, blood potassium levels decreased below baseline levels (3.83 ± 0.15 mmol × L^–1^) to between 3.55 ± 0.28 and 3.22 ± 0.13 (*p* < 0.0005 vs. Pre; Figure 3D). By contrast, saline infusion increased blood potassium levels from the same time point, to between 4.07 ± 0.4 and 4.14 ± 0.41 mmol × L^–1^ (*p* < 0.0011 vs. Pre; Figure 3D), with levels having returned to baseline at the end of the trial (*p* = 0.1067; 180 min vs. Pre). The lactate infusion also lowered blood HCT levels to 36.0 ± 3.02% (*p* < 0.0001 vs. Pre; Figure 3E), partly recovering to 38.0 ± 3.36% during the subsequent recovery period (*p* = 0.1044 vs. Pre; Figure 3E). Blood HCT levels remained unchanged in the Saline trial (*p* > 0.3623 vs. Pre). Serum cortisol levels fell between 19- and 30% during the lactate and saline infusion, respectively (*p* < 0.0241 vs. Pre; Figure 3F). In the Saline trial, cortisol levels remained depressed throughout the recovery (*p* < 0.0002 vs. Pre), while cortisol returned to baseline levels in the Lactate trial at 15- and 30 min post infusion (*p* > 0.2615). At the same time-points, a difference between the trials was evident (min 75 and 90; *p* < 0.0159 for Lactate vs. Saline; Figure 3F).

### Pro and mBDNF levels

**FIGURE 4:**
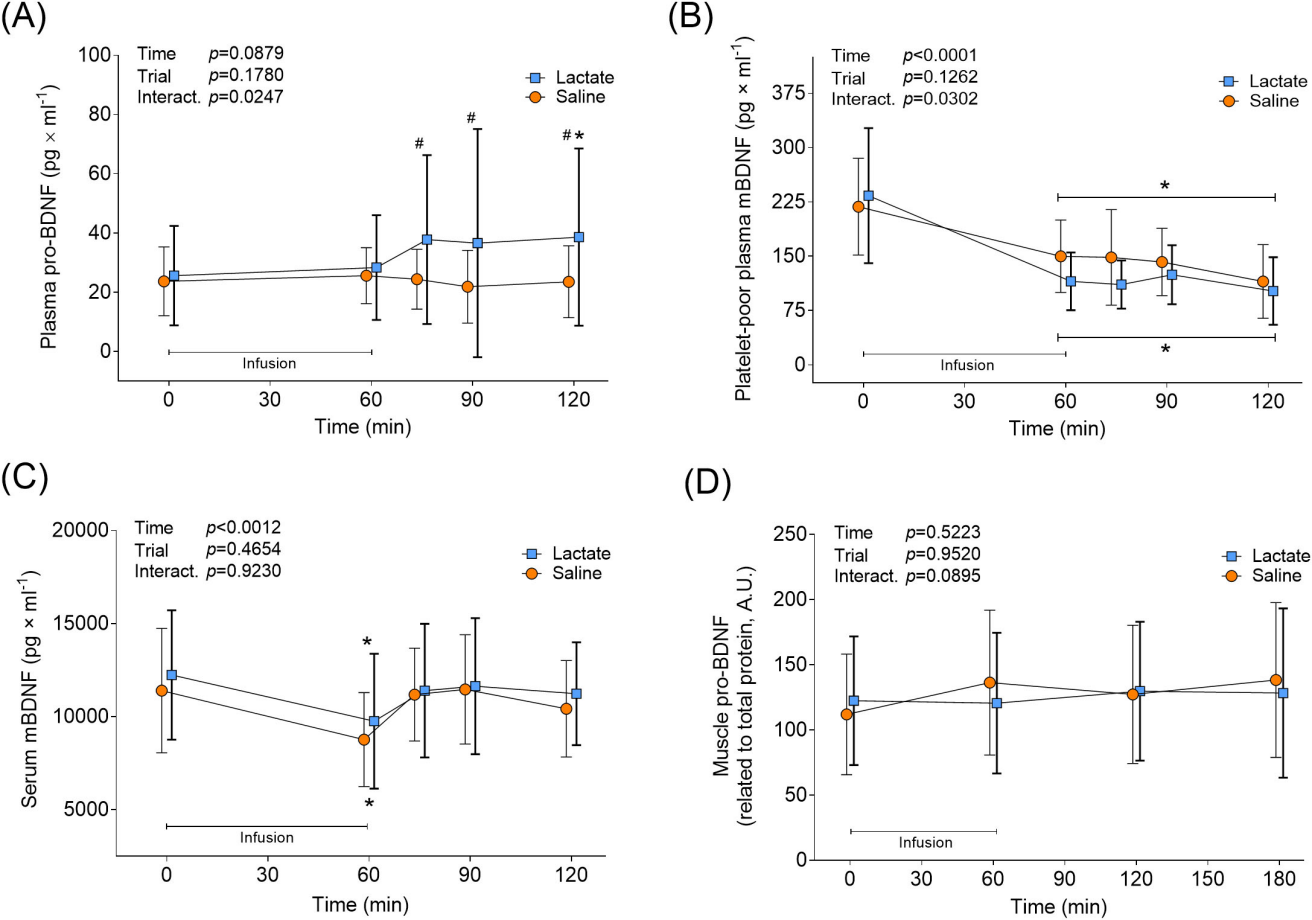
BDNF levels in circulation and muscle in response to lactate infusion. **(A)** Plasma pro-BDNF, **(B)** platelet-poor plasma mBDNF and **(C)** serum mBDNF levels throughout the Lactate and Saline trial (*n* = 12 vs. 12). **(D)** Muscle pro-BDNF levels (*n* = 12 vs. 6). Muscle mBDNF was non-detectable. A.U., arbitrary units. *p < 0.05 vs. Pre, #*p* < 0.05 vs Saline. Two-way repeated measures ANOVA, Tukey’s HSD *post hoc*. Error bars show variability as ± 1 standard deviation (SD). Error bars for the lactate trial are shown in bold.

In the Lactate trial, plasma pro-BDNF levels were unaltered during the infusion, but increased by 50% from 25.58 ± 16.6 to 38.58 ± 30.0 pg × ml^–1^ an hour after infusion cessation (min 120 vs. Pre, *p* = 0.0361; Figure 4A). Plasma pro-BDNF levels were higher during the recovery period in the Lactate trial compared to the Saline trial (min 75-, 90 and 120; *p* < 0.0286; Figure 4A). Pro-BDNF levels remained unchanged in the Saline trial (*p* > 0.9999 vs. Pre; Figure 4A). Both infusion protocols affected PPP mBDNF levels similarly, with a distinct 31%–51% decrease from baseline levels seen at the end of the infusion (*p* < 0.0002 vs. Pre; Figure 4B). *Post hoc* analysis revealed no time-point specific differences between trials, despite a significant interaction effect (*p* = 0.0302; Figure 4B). The two infusion protocols also had a similar effect on serum mBDNF levels, with levels decreasing by 20%–23% from baseline to the end of infusion (*p* < 0.0014 vs. Pre; Figure 4C). Instead of remaining decreased during the recovery from infusion, as noticed for plasma mBDNF levels, serum mBDNF levels returned to baseline 15 min after infusion cessation and stayed there until trial end (*p* > 0.4932 vs. Pre for Lactate and Saline trial; Figure 4C). Neither infusion protocol affected muscle levels of pro-BDNF, as it remained unchanged during the infusion- and recovery period (*p* > 0.0895 for Main- and Interaction effects; Figure 4D). Muscle levels of mBDNF were non-detectable with immunoblotting (data not shown).

### Fiber type and BDNF Val66Met polymorphism

**FIGURE 5:**
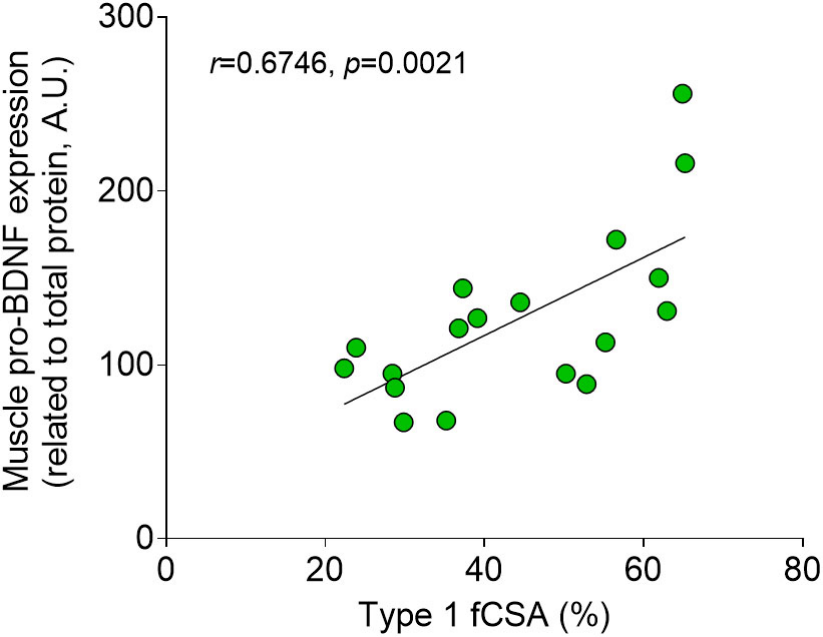
Fiber type and muscle pro-BDNF expression. Correlation with Pearson’s *r* (*n* = 18) between type I percentage cross-sectional area (fCSA%) and muscle pro-BDNF expression (A.U.).

A significant positive correlation was seen between muscle pro-BDNF expression and type I fCSA% (*n* = 18, *r* = 0.6746, *p* = 0.0021, Figure 5). Genotyping results showed allele-specific bands for two genotypes, Val/Val and Val/Met, with no participant showing the mutation in both alleles (Met/Met). The BDNF Val66Met polymorphism was expressed in 7 out of 18 participants (39%), with a similar distribution in genotype seen within the experimental-and control group (42 and 33% respectively, Table 1). No differences in pre- or delta BDNF values were detectable between participants with and without the Val66Met polymorphism in the experimental group (*n* = 12, *p* > 0.05) for any of the BDNF variables. Likewise, when dividing all participants (*n* = 18) based on BDNF genotype, no difference was seen in muscle pro-BDNF levels between Val/Val- and Val/Met groups (data not presented).

## Discussion

In the present work, we elevated blood lactate levels in humans at rest through venous infusion to levels that are normally attained during moderate- to high-intensity exercise. We asked if lactate in isolation could affect circulating levels of pro- and mBDNF, as well as muscle pro-BDNF levels. We further investigated whether muscle fiber type distribution, genotype and cortisol had an impact on the collective BDNF levels. Our main findings were: (1) the elevated lactate levels had no influence on plasma- or serum levels of mBDNF or muscle levels of pro-BDNF, and (2) the lactate infusion resulted in elevated plasma levels of pro-BDNF.

Here, plasma mBDNF levels were unaffected by increased systemic lactate, which partly contrasts with previous studies in rodents and humans (El Hayek et al., 2019; Edman et al., 2024). After acknowledging the difference with the lack of exercise stimuli in the present study, a plausible explanation for the discrepancy may be the relatively modest systemic lactate concentrations achieved by our protocol, peaking at approximately 5.9 mmol/L in plasma before a rapid decline (Figure 2B). This level may have been insufficient to stimulate mBDNF release. For instance, El Hayek et al. (2019) reported that lactate administration in resting mice resulted in much higher blood lactate concentrations (13–20 mmol/L); however, they did not measure acute mBDNF levels. In line with the idea of insufficient lactate levels attained, Schiffer et al. (2011) showed that an aggressive venous sodium lactate infusion in humans at rest, resulting in plasma lactate of approximately 12 mM, evoked a rapid and short-lived increase in serum mBDNF levels. Provided that this rapid effect could not be due to *de novo* production and considering the findings of Moberg et al. (2024), the source for the elevated circulating mBDNF was most likely peripheral tissue. Moreover, it is highly important to distinguish between acute and long-term changes in BDNF levels in response to elevated lactate. In Moberg et al. (2024), lactate injections did not alter BDNF expression or protein content in the acute phase. El Hayek et al. (2019) administered repeated lactate injections in mice that resulted in elevated hippocampal levels of mBDNF after 30 days. It is thus plausible that lactate infusion in our case, and in Moberg et al. (2024), stimulated brain BDNF production, which could have resulted in measurable elevated brain BDNF levels with weeks of repeated increases in systemic lactate.

While skeletal muscle levels of mBDNF were non-detectable, we clearly detected pro-BDNF, both in line with previous results (Edman et al., 2024). The lactate infusion significantly increased muscle lactate levels in the present study, however, pro-BDNF levels were unaffected (Figure 4D). This is consistent with what is observed when lactate is infused during exercise (Edman et al., 2024). Here, muscle lactate levels peaked at approximately 15 mmol/kg dry wt (3.98 mmol/L i.c. water) in the Lactate trial, while the levels attained in our previous investigation were twice that in both the exercise-only and exercise lactate trials (27- and 32 mmol/kg dry wt. or 6.20- and 7.25 mmol/L i.c. water, respectively) (Edman et al., 2024), suggesting that it was not a question of inadequate intramuscular lactate levels in the present study. In Edman et al. (2024) exercise resulted in augmented muscle pro-BDNF levels with no additional increase when lactate levels were further elevated by infusion. It could thus be argued that muscle contractions *per se*, and not lactate, are the stimulator of elevated muscle pro-BDNF levels. On the other hand, it might be possible that we could not detect an increase in muscle pro-BDNF because there was a simultaneous release of the pro-protein into the circulation while it was being synthesized. This would support the argument that the skeletal muscle is a contributing source for the elevated blood levels of pro-BDNF in the present study. This is also supported by our recent finding of a skeletal muscle release of pro-BDNF after high-intensity exercise (Tarassova et al., 2025).

Considering the systemic lactate levels reached in earlier studies (El Hayek et al., 2019; Edman et al., 2024; Schiffer et al., 2011), it can be argued that we should have set out to attain higher blood lactate levels. However, a more aggressive infusion protocol would have resulted in substantial blood alkalosis (pH > 7.45) and thereby limited the physiological relevance of the intervention. Also, the venous sodium lactate infusion lowered blood levels of potassium (Figure 3D), and a higher dose would have increased the risk for acute hypokalemia. Even with the current dose, some individuals were provided with oral potassium citrate after the completed protocol to prevent hypokalemia (potassium < 3 mmol/L). Altering one physiological variable in humans *in vivo* without significantly changing others is challenging and poses a clear limitation with the model utilized here. Even with the moderate infusion protocol used, the sodium lactate infusion still resulted in a distinct increase in pH (Figure 3A). Hence, there is reason to acknowledge the potential influence of pH on plasma and serum mBDNF levels in this study. However, considering that we previously detected increased plasma mBDNF levels with a similar increase in pH (Edman et al., 2024), it is unlikely that the increased pH attained here eliminated the potential effect of lactate on mBDNF levels.

Conversely, we believe that the lack of concomitant exercise stimuli in the present study may strongly contribute to the absence of an mBDNF response in plasma. Exercise typically induces lactate production primarily within skeletal muscle, which then acts as a net exporter of lactate into the circulation (Brooks, 2018). During exogenous lactate infusion at rest, however, skeletal muscle becomes a net consumer of lactate (van Hall et al., 2009). This altered metabolic context may reduce lactate uptake by the brain, given that skeletal muscles, particularly oxidative type I fibers with their higher MCT content (Juel and Halestrap, 1999), compete for circulating lactate. The high density of MCTs in oxidative type I fibers might facilitate efficient lactate shuttling into the cells, potentially diverting lactate away from the brain and thereby limiting the brain’s ability to induce mBDNF production and/or release. The latter is the parameter that logically could be discussed here. It must be stated that brain BDNF production cannot, for obvious reasons, be assessed in a human model, and circulating levels or brain AV-differences are used as surrogate measures for brain BDNF metabolism. Assessing both plasma and serum mBDNF, we recently found no acute brain mBDNF release in relation to different exercise intensities in trained humans (Tarassova et al., 2025). Although exercise could have stimulated brain BDNF production in that setting, this was not accompanied by a release into the circulation. This would argue that the acute increase in circulating BDNF with exercise derives from the skeletal muscle and exercise-induced stimulation of platelets, and that circulating mBDNF is a better surrogate for long-term changes in brain mBDNF levels (Klein et al., 2011).

The shift in lactate dynamics may also explain why we observed increased plasma pro-BDNF levels after lactate infusion, despite the lack of a corresponding rise in mBDNF. Here we confirm the fiber type-specific pro-BDNF expression first demonstrated by Edman et al. (2024) (Figure 5). The preferential lactate uptake by oxidative type I muscle fibers, known for their high concentrations of pro-BDNF (Edman et al., 2024), could lead to an increased release of pro-BDNF into the circulation. This mechanism could mimic that observed during exercise (Tarassova et al., 2025). Moreover, given the intra- and extracellular cleavage capacity (Kojima et al., 2019), pro-BDNF could potentially be regarded as an mBDNF precursor pool. According to this argument, elevated circulating levels of pro-BDNF in this case would logically have led to elevated mBDNF. The lack of detecting such a relationship here can be explained by delayed cleavage dynamics, the markedly lower levels of pro-BDNF compared to mBDNF here, as well as by a potential skeletal muscle uptake of mBDNF as described after exercise by Tarassova et al. (2025).

We observed that both infusion protocols affected plasma mBDNF similarly, with levels dropping significantly from the end of infusion, compared to baseline (Figure 4B). This may be due to the proposed diurnal rhythmicity of BDNF, with plasma levels suggested to peak in the morning and subsequently fall throughout the day (Begliuomini et al., 2008), combined with the plasma volume expansion noted in both infusion protocols (Figure 3E). In the Saline trial, similarly to what was reported by Begliuomini et al. (2008), we also observed both cortisol- and mBDNF levels decreasing concomitantly throughout the 2-h sampling period (Figures 3F, 4B), however, neither absolute levels nor changes in cortisol were correlated (data not shown). Although cortisol levels decreased in the Lactate trial, the reduction was less pronounced. Furthermore, at 15 and 30 min after the end of the infusion, cortisol levels were higher in the Lactate trial compared to the Saline trial (Figure 3F). Although BDNF and cortisol have been described as interplaying in the trophic regulation of brain cells (de Assis and Gasanov, 2019; Tsimpolis et al., 2024), the functional effect of this crosstalk on acute circulating BDNF levels in response to exercise and lactate, as well as lactate in isolation, is to our knowledge, unknown.

In the past two decades, a significant degree of attention has been directed to the single nucleotide polymorphism causing a valine to methionine substitution at codon 66, i.e., Val66Met. This after the seminal paper by Egan et al. (2003), which illustrated that the Val66Met substitution in the protein negatively alters BDNF release and cellular trafficking and thus reduces hippocampal function. In line with this, it has been suggested that Val66Met-carriers have an augmented risk for depression and cognitive decline (Liu et al., 2020; Zarza-Rebollo et al., 2024). This notion argues that Val66Met-carriers would exhibit a reduced exercise-induced circulatory BDNF release, but evidence in support of this is at present inconclusive. On the contrary, acute exercise-induced improvements in cognitive function have been shown to be unaffected by BDNF polymorphism, but to correlate with exercise-induced lactate levels (Ballester-Ferrer et al., 2022). This is in line with the present data, where basal and delta pro-/mBDNF levels were similar between Val/Val and Val/Met carriers, but elevated lactate levels increased circulating pro-BDNF levels irrespective of genotype (data not shown).

Based on the previous literature, together with the present findings, we propose the following perspective for the exercise-BDNF-brain-function relationship in humans. Since numerous studies have demonstrated acute increases in circulating BDNF with exercise in humans (Dinoff et al., 2017), this has been incautiously and sometimes wrongly interpreted and accepted as evidence for increased *de novo* synthesis and release of BDNF from the CNS. Although increased BDNF released from the brain has been demonstrated acutely with exercise (Rasmussen et al., 2009; Seifert et al., 2010), this has recently been questioned (Tarassova et al., 2025). Importantly, one should remember that there is no evidence suggesting that an elevated brain BDNF release is associated with elevated brain BDNF levels or improved brain function. Also, an increased *de novo* synthesis of BDNF cannot be significantly detected either in the brain or in the circulation within minutes or hours after acute exercise, which is the common finding for elevated circulating levels in humans. While higher exercise intensity, and thus consequently higher lactate levels, are associated with higher acute circulating levels of BDNF (Ferris et al., 2007; Marston et al., 2017; Reycraft et al., 2020), we strongly propose that this is due to exercise-induced physiological perturbations and/or lactate stimulating peripheral BDNF release and platelet activation. Based on the present findings and those in Tarassova et al. (2025) and Edman et al. (2024), we suggest that acutely elevated systemic lactate stimulates a release of pro-BDNF from skeletal muscle, predominantly from type I muscle fibers. Exercise with higher intensity, and thus higher lactate, stimulates a more profound mobilization of mBDNF in the circulatory system, which might be relevant for the peripheral nervous system and tissue repair, but of little relevance for brain function. Importantly, we regard high-intensity exercise training, with lactate as a key mediator, as an important mechanism for BDNF synthesis in the brain and brain function in the long term. However, researchers should refrain from extrapolating acute circulatory BDNF levels after exercise to positive brain adaptations long term. We propose that future human research should be directed to the importance of exercise-induced lactate-BDNF for peripheral nervous system function, as well as devotion to methods that can assess BDNF levels or effects directly in the CNS after an acute intervention.

In this paper, we employed a human *in vivo* model to investigate the effect of increased systemic lactate levels at rest on circulating levels of both pro- and mBDNF isoforms. Despite reaching markedly higher lactate levels during the 1-h infusion, mBDNF remained unaltered in both plasma and serum. By contrast, lactate infusion increased plasma pro-BDNF levels, suggesting a differential regulation of the two BDNF isoforms in response to lactate when there is no simultaneous exercise stimulus. The present data support the notion that lactate is a molecule involved in the regulation of BDNF metabolism in humans.

## Data Availability

The raw data supporting the conclusions of this article will be made available by the authors, without undue reservation.
